# Cellular and Molecular Signaling Meet the Space Environment

**DOI:** 10.3390/ijms24065955

**Published:** 2023-03-22

**Authors:** Khaled Y. Kamal, John M. Lawler

**Affiliations:** 1 Redox Biology & Cell Signaling Laboratory, Department of Kinesiology & Sport Management, Texas A&M University, College Station, TX 77843, USA; jml2621@tamu.edu; 2 Department of Nutrition, Texas A&M University, College Station, TX 77843, USA

During space missions that travel beyond the cocoon of the Earth’s magnetosphere, astronauts are subjected to the microgravity and radiation stressors of outer space. Understanding and mitigating the adverse effects of microgravity and space radiation impose profound challenges for future missions to Mars and back and future Lunar missions. In recent years, cellular and molecular signaling in space biology has become a vital research topic. Therefore, we are pleased to present this Special Issue entitled “Cellular and Molecular Signaling Meet the Space Environment,” which delves into the significant advances and challenges that face research in space biology and the life sciences community.

Microgravity and space radiation experienced during spaceflight, especially beyond low Earth orbit (LEO), clearly affect cellular and molecular signaling pathways, leading to alterations in cell signaling [1], remodeling [2], repair, cell cycle [3], and thus, functional changes. Therefore, understanding the cellular and molecular signaling mechanisms that occur in space is crucial to developing countermeasures that mitigate the harmful effects of spaceflight on living organisms and how they are able to adapt themselves. This Special Issue, “Cellular and Molecular Signaling Meet the Space Environment,” presents a collection of research articles, reviews, and perspectives showcasing the latest cellular and molecular signaling developments in space biology, ranging from rodent and cell cultures to human studies. The articles in this Special Issue cover a wide array of topics, including the impact of space gravitational changes on epigenetic modifications on the immune system, the molecular mechanisms underlying skeletal muscle atrophy, and the osteoblast differentiation that induces bone loss. Furthermore, select articles investigating human health during spaceflight, including research on understating muscle deconditioning and other reviews on gynecology and cancer in female astronauts, are included. Thus, the importance of this Special Issue lies within its diversity of topics that include space biology, countermeasure development, the impact on space missions, and clinically relevant studies.

In this Special Issue, the molecular mechanisms of gravitational alterations using rodents and cell culture models have been covered. One of the articles in this issue focuses on the impact of hypergravity exposure on post-translational histone modifications in the thymus of mice [4]. Calcagno and his colleagues present evidence that hypergravity exposure downregulates lysine 27 trimethylation (H3K27me3) in the thymus, which affects the Vβ germline transcript expression before V(D)J recombination, contributing to the alteration of the TCR repertoire [4]. This study implicates the histone methyltransferase PRC2 complex of EZH2 (Enhancer of Zeste Homolog) in regulating the T-cell receptors heavy chain (TCRβ) locus chromatin structure, highlighting a potential mechanism for how gravity changes affect the immune system [4]. Another article in this Special Issue identified the role of simulated microgravity (SMG) on osteoblast differentiation (OBD) and induced bone loss through the Wnt/β-catenin pathway, though the underlying mechanism is unknown. Fan et al. [5] reported that FAK mediates SMG-induced changes to Wnt/β-catenin in mouse osteoblasts, leading to reduced bone formation. The authors suggest that targeting FAK signaling could be a new therapeutic approach for preventing bone loss in astronauts and osteoporotic patients.

Furthermore, Blottner et al. [6] conducted a study to investigate the expression pattern of Homer short and long isoforms in the skeletal muscles of male C57Bl/N6 mice after 30 days in space compared to ground-housed animals and rats subjected to hindlimb unloading. The results showed an increase in Homer1a transcripts and the downregulation of Homer2 long-form in the soleus muscle of animals exposed to microgravity or hindlimb unloading, suggesting the possible role of Homer isoforms in neuromuscular junction imbalance/plasticity. The reduced Homer crosslinking at the NMJ consequent to increased Homer1a and/or reduced Homer2 may contribute to muscle-type specific atrophy resulting from microgravity and HU disuse suggesting mutual mechanisms.

From clinical aspects, Guilhot et al. [7] reported that the loss of muscle mass and strength, as well as increased intermuscular adipose tissue (IMAT), is a recognized consequence of muscle deconditioning experienced in prolonged microgravity. This human clinical study used the dry immersion (DI) model, which led to severe muscle deconditioning, to investigate the effects of fast and severe muscle disuse on fibro-adipogenic progenitor fate and behavior. The results suggest that altered extracellular matrix structure and signaling pathways occur early during DI, favoring fibro-adipogenic progenitor differentiation into adipocytes.

In addition to research articles, this Special Issue includes review articles and perspectives that provide a broader perspective on cellular and molecular contributions to health hazards from deep space missions. One such article discusses the hazards of outer space, particularly the impact of ionizing radiation and microgravity on the health of astronauts, with a focus on the increased risk of gynecological cancer in women. Women historically have been permitted to spend less time in space due to their higher incidence of radiation-induced cancers. The aim of the article is to provide a summary of existing research and identify further studies that are needed to ensure safer space exploration and better post-flight screening and management for women astronauts [8].

Overall, this Special Issue presents a comprehensive overview of the current state of research that explores the cellular and molecular mechanisms that underlie space biology (Table 1). The articles cover a wide range of topics and provide valuable new insights into the complex cellular and molecular signaling that occurs in space. In conclusion, we believe that this Special Issue will be of significant interest and utility to researchers and students working in the field of space life science. We hope that the research presented in this issue will stimulate continued research in the field and contribute to the development of effective countermeasures that will protect the safety and well-being of astronauts during spaceflight.

## Figures and Tables

**Table 1:** Summary of the IJMS Special Issue.

Subject	Space Platform	Cellular Mechanisms	References
C57BL6/J mice, Cell culture (SCIET27)	*Hypergravity* Large radius centrifuge (radius of 1.80 m)	Hypergravity downregulates post translational histone modifications (H3K27me3) at the TCRβ locus with the implication of EZH2 in the regulation of TCRβ in T lymphopoiesis.	Calcagno, Ouzren, Kaminski, Ghislin and Frippiat [4]
Mouse osteoblast cell line (MC3T3-E1)	*Microgravity* Random positional machine (RPM) Hindlimb unloading (HU)	Simulated microgravity downregulated FAK signaling, Wnt/β-catenin, SMG-affected tibial trabecular bone loss, suggesting the key role of FAK signaling for astronauts at risk of bone loss.	Fan, Wu, Cooper, Magnus, Harrison, Eames, Chibbar, Groot, Huang and Genth [5]
C57BL/N6 male mice	*Microgravity* Spaceflight (ISS), Hindlimb Unloading (HU)	Microgravity upregulated reciprocal Homer1a and Homer2 isoform expression induced skeletal muscles atrophy during neuromuscular junction imbalance/plasticity in space.	Blottner, Trautmann, Furlan, Gambara, Block, Gutsmann, Sun, Worley, Gorza and Scano [6]
Human subjects	*Muscle deconditioning* Dry Immersion (DI) model	Using a dry immersion model which led to muscle deconditioning (model for sever muscle wasting) through altered extracellular matrix structures, signaling pathways. This model induced fibro-adipogenic progenitor differentiation into adipocytes	Guilhot, Fovet, Delobel, Dargegen, Jasmin, Brioche, Chopard and Py [7]
